# Select Bcl-2 antagonism restores chemotherapy sensitivity in high-risk neuroblastoma

**DOI:** 10.1186/s12885-016-2129-0

**Published:** 2016-02-13

**Authors:** Rachel Tanos, Dipan Karmali, Srilatha Nalluri, Kelly C. Goldsmith

**Affiliations:** Division of Hematology/Oncology, Aflac Cancer and Blood Disorders Center, Children’s Healthcare of Atlanta, Atlanta, GA 30322 USA; Department of Pediatrics, Emory University School of Medicine, Atlanta, GA 30322 USA

**Keywords:** Bcl-2 homology proteins, Bcl-2 antagonist, ABT-199, Apoptosis, Neuroblastoma

## Abstract

**Background:**

Pediatric patients with high-risk neuroblastoma (HR NB) often fail to respond to upfront intensive multimodal therapy. Tumor-acquired suppression of apoptosis contributes to therapy resistance. Many HR NB tumors depend on the anti-apoptotic protein Bcl-2 for survival, through Bcl-2 sequestration and inhibition of the pro-apoptotic protein, Bim. Bcl-2 dependent xenografts derived from aggressive human NB tumors are cured with a combination of cyclophosphamide and ABT-737, a Bcl-2/Bcl-X_L_/Bcl-w small molecule antagonist. The oral analogue to ABT-737, Navitoclax (ABT-263), clinically causes an immediate drop in peripheral platelet counts as mature platelets depend on Bcl-x_L_ for survival. This led to the creation of a Bcl-2 selective inhibitor, ABT-199 (Venetoclax). A Phase I trial of ABT-199 in CLL showed remarkable antitumor activity and stable patient platelet counts. Given Bcl-X_L_ does not play a role in HR NB survival, we hypothesized that ABT-199 would be equally potent against HR NB.

**Methods:**

Cytotoxicity and apoptosis were measured in human derived NB cell lines exposed to ABT-199 combinations. Co-Immunoprecipitation evaluated Bim displacement from Bcl-2, following ABT-199. Murine xenografts of NB cell lines were grown and then exposed to a 14-day course of ABT-199 alone and with cyclophosphamide.

**Results:**

Bcl-2 dependent NB cell lines are exquisitely sensitive to ABT-199 (IC_50_ 1.5–5 nM) in vitro, where Mcl-1 dependent NBs are completely resistant. Treatment with ABT-199 displaces Bim from Bcl-2 in NB to activate caspase 3, confirming the restoration of mitochondrial apoptosis. Murine xenografts of Mcl-1 and Bcl-2 dependent NBs were treated with a two-week course of ABT-199, cyclophosphamide, or ABT-199/cyclophosphamide combination. Mcl-1 dependent tumors did not respond to ABT-199 alone and showed no significant difference in time to tumor progression between chemotherapy alone or ABT-199/cyclophosphamide combination. In contrast, Bcl-2 dependent xenografts responded to ABT-199 alone and had sustained complete remission (CR) to the ABT-199/cyclophosphamide combination, with one recurrent tumor maintaining Bcl-2 dependence and obtaining a second CR after a second course of therapy.

**Conclusion:**

HR NB patients are often thrombocytopenic at relapse, raising concerns for therapies like ABT-263 despite its HR NB tumor targeting potential. Our data confirms that Bcl-2 selective inhibitors like ABT-199 are equally potent in HR NB in vitro and in vivo and given their lack of platelet toxicity, should be translated into the clinic for HR NB.

## Background

Tumor cells depend on the suppression of apoptosis to survive the multitude of stressors they encounter on a daily basis, such as genetic mutations, hypoxia, chemotherapy and radiation. Chemotherapy induces the intrinsic pathway of apoptosis that is tightly regulated by mitochondrial resident Bcl-2 family proteins whose structure is comprised of one to four highly conserved Bcl-2 homology (BH) domains [1]. Within this family Bcl-2 proteins with multiple BH domains can be either anti-apoptotic (Bcl-2, Bcl-X_L_, Bcl-w, Mcl-1, Bfl-1/A1) or pro-apoptotic (Bax, Bak) and a subset containing only the BH3 domain are all pro-apoptotic (Bim, Bid, Puma, Bad, Bik, etc.) [2]. The BH3 domain forms an amphipathic alpha helix that fits snuggly into the hydrophobic groove of multidomain members, leading to the specific binding of certain BH3 proteins to select multidomain members. Tumor cells often increase the expression and/or activity of anti-apoptotic Bcl-2 members to bind to and sequester stress-activated BH3 proteins to prevent their binding to Bax and or Bak to inhibit apoptosis. For example, a cancer dependent on Bcl-2 for survival, such as chronic lymphocytic leukemia (CLL), has an abundance of Bcl-2 that actively sequesters the BH3 protein, Bim, making the cancer cell “primed to die” in the event that other cellular stressors activate additional BH3 proteins to overcome the Bcl-2 binding capacity within the cell [3].

Knowledge of the unique primed state of cancer cells led to the development of novel classes of pro-apoptotic small molecules that disrupt critical Bcl-2 family protein:protein interactions to reinstate apoptosis (e.g., HA14-1 [4], AT-101 [5], ABT-737 [6], ABT-263 [7]). The most potent candidates designed to antagonize Bcl-2, Bcl-X_L_ and Bcl-w includes ABT-737 and its clinical homologue, ABT-263 (Navitoclax, Abbvie) [6, 8]. ABT-737 mimics the BH3 protein Bad and targets the hydrophobic pocket of Bcl-2, Bcl-X_L_ and Bcl-w with low nanomolar affinity.

Navitoclax has been tested in clinical trials for adult cancers like CLL, AML and Small Cell Lung Carcinoma [9, 10]. Navitoclax treatment in these trials caused an immediate drop in the patient’s peripheral platelet count, potentially putting them at increased risk for bleeding. This dose limiting toxicity of Navitoclax is caused by its Bcl-X_L_ -targeting properties as mature circulating platelets depend on Bcl-X_L_ for survival [11, 12]. Recently, a more selective Bcl-2 antagonist also from Abbvie, ABT-199 (Venetoclax), became clinically available [13]. When tested in a phase 1 trial for adult CLL, ABT-199 showed remarkable tumor killing effects, causing overwhelming tumor lysis due to its potent on target disruption of Bcl-2 and its binding partners [13]. ABT-199 selectively targets the hydrophobic pocket of Bcl-2 and not Bcl-X_L_, safeguarding against unacceptable platelet effects [14]. Given these Bcl-2 select antagonists are well tolerated and performing well in adult cancer trials, they have high potential to translate to the pediatric setting to treat Bcl-2 dependent tumors. Here we provide evidence that this holds true in a highly lethal pediatric solid tumor known to have functional dependence on Bcl-2, neuroblastoma.

Patients with high-risk neuroblastoma (HR NB) initially respond to treatment yet > 50 % still die of disease due to the emergence of chemotherapy resistance [15]. We and others have shown that chemotherapy induced apoptosis is inhibited in NB due to alterations in Bcl-2 family protein interactions at the mitochondria [16]. Bcl-2 dependence in NB, like other cancers, is not due to an overabundance of Bcl-2 in the cell compared to other anti-apoptotic members, but due to the functional dependence on Bcl-2 to bind to and sequester activated Bim [17]. Differences in post-translational modifications of Bim seem to play a part in determining its selectivity to Bcl-2 over Mcl-1 in HR NB [18]. We have shown that ABT-737 in combination with cyclophosphamide, induces a complete durable tumor response in mice bearing large xenografts of even the most therapy resistant NB cell lines, including those with MYCN amplification and ALK mutations [19]. Functional characterization of Bcl-2 dependence patterns by co-immunoprecipitation (co-IP) confirmed that ABT-737 sensitive NB cell lines depend on Bcl-2 and not on Bcl-X_L_ to sequester Bim for survival, making a large subset of NBs prime candidates for select Bcl-2 antagonism [19].

Thus our work and others supports a role for the functional identification and targeting of Bcl-2 mediated resistance mechanisms in HR NB. We have tested relapsed NB cell lines as well as primary human high-risk NB tumors and validated that similar Bcl-2 dependence patterns exist in relapsed as well as primary tumors [19]. In order to facilitate a rapid translation of ABT-199 into clinical trials for patients with high-risk NB, we demonstrate with these studies that ABT-199 is just as potent pre-clinically as the Bcl-2/ Bcl-X_L_/Bcl-w antagonists that preceded it.

## Methods

### Cell lines

Neuroblastoma cell lines with MYCN amplification **(**IMR-5, SMS-SAN and NB-1643) were used (courtesy of COG Cell Line Repository and Michael D. Hogarty, MD, Laboratory, CHOP). Neural cells were grown in RPMI-1640 (Life Technologies) supplemented with 10 % fetal bovine serum, 2 mM L-Glutamine, 100 U/mL of penicillin. Tissue culture was at 37 °C in a humidified atmosphere of 5 % CO_2_. All cell lines were validated as unique using STR based genotyping and confirmed using the COG cell line STR genotype database (www.cog.org).

### Cell viability assays

2 × 10^4^ NB cells/well were plated in triplicate in 96-well plates in phenol red-free media and allowed to adhere for 24 h. Cells were then treated with ABT-199, doxorubicin, melphalan or vehicle controls. After 48 h, WST-1 reagent (Roche) was added and absorbance at 590 nM was recorded and normalized to the background. Error bars represent the average of the three technical triplicates and are representative of results seen in two separate biologic experiments.

### PARP cleavage

Cells were treated with the indicated ABT-199 doses for 24 h. Cells were lysed with CHAPS buffer (10 mmol/L HEPES, 150 mmol/L NaCl, 2 % CHAPS [Sigma Aldrich, MO]) as previously described [19]. Protein from cell lysate (25 μg per well) was loaded and an anti-PARP antibody (Cell Signaling, MA; #9542) was used to detect PARP cleavage.

### Caspase 3/7 assay

2 × 10^4^ cells/well were plated in a 96-well plate and treated with the appropriate dose of ABT-199 or Etoposide (Sigma Aldrich, MO) for 24 h. Caspase-Glo 3/7 Assay kit (Promega, WI; G8090) was used to quantify caspase activity according to the manufacturer’s specifications**.** Error bars represent the average of three separate biologic experiments.

### Annexin/PI apoptosis assay

NB cells were treated with 5 nM ABT-199 or 10 μM etoposide for 24 h. Following the incubation, cells were harvested, washed twice with PBS and re-suspended in Binding Buffer (8 g NaCl, 0.2 g KCl, 1.44 g Na_2_HPO _4_•7H_2_0, 0.24 g KH_2_PO_4_) at a concentration of 1 × 10^6^ cells/mL. Cells were incubated with Annexin V-PE and 7-AAD (BD Biosciences, CA) for 15 min and then analyzed immediately by flow cytometry using the FACSCanto II flow cytometer (BD Biosciences, CA). Histograms represent percentage of cells found to be Annexin V positive. Error bars represent the average of two separate experiments.

### Co-immunoprecipitation

Cells were lysed with CHAPS buffer with 300 μg of protein lysate added to antibody-matrix complex as previously described [18]. Xenograft tumor sample were harvested and immediately dissociated though a 0.45 μm sterile filter, lysed, and immunoprecipitated as above. Antibodies used as previously described [19].

### Murine xenograft studies

Xenografts (IMR5 and NB-1643) were established subcutaneously in the flank of 4–6 week old nu/nu athymic mice (Jackson Laboratories, ME) as described [19]. When tumor volume reached 150 to 200 mm^3^, mice were treated either (i) by intraperitoneal (IP) injection of cyclophosphamide (CPM: 75 mg/kg) or saline solution IP twice weekly for 2 weeks, (ii) by oral gavage of ABT-199 (100 mg/kg/day for 2 weeks) or vehicle solution, (iii) by a combination of CPM with ABT-199 at monotherapy dosing/duration. ABT-199 was formulated in a mixture of 60 % Phosal 50 PG, 30 % PEG 400, and 10 % Ethanol. One animal from the combination treatment was retreated with the same therapy schedule when the recurrent tumor reached 200 mm^3^. Tumor volumes were evaluated using calipers and calculations using the ellipsoid formula (Length × width^2^ × 0.52). Animals were sacrificed when tumor volumes exceeded 2000 mm^3^, according to the Emory Institutional Animal Care and Use Committee Guidelines. One mouse with recurrent tumor from the combination treatment was sacrificed for tumor co-immunoprecipitation studies. Mice were housed in a temperature- and light-controlled pathogen-free facility and given access to sterilized food and water ad libitum. All animal work abided by and was carried out under a protocol approved by the Emory Institutional Animal Care and Use Committee.

### Statistical evaluation

Survival analysis were conducted according to the method of Kaplan-Meier and analyzed by a Mantel-Cox test. GraphPad Prism software was used to determine statistical significance between treatments and the IC_50_ in the cell survival analyses. *P*-values < 0.05 were considered statistically significant.

## Results

### Select Bcl-2 antagonism potently activates apoptosis in HR NB cell lines in vitro

**Fig. 1:**
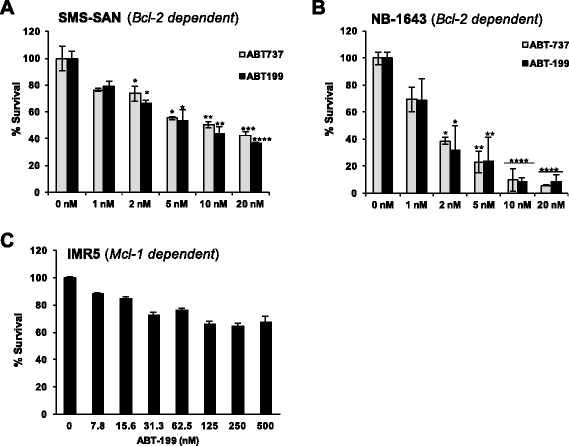
Bcl-2 dependent NB cell lines are sensitive to ABT-199 in vitro. Bcl-2 dependent cell lines SMS-SAN (**a**), NB-1643 (**b**) were exposed to ABT-737 or ABT-199 at given concentrations for 48 h and then evaluated for survival by WST-1. Mcl-1 dependent (**c**) IMR5 was completely resistant to Bcl-2 antagonism. Values statistically significant from 0 nM control are denoted with stars as follows: (*) *p* ≤ 0.05, (**) *p* ≤ 0.01, (***) *p* ≤ 0.001, (****) *p* ≤ 0.0001, as determined by student’s t test

NB cell lines previously characterized for different Bcl-2 family dependence patterns [19] were challenged with identical concentrations of either ABT-199 or ABT-737 and assessed for effects on survival. Our past evaluation of Bim:Bcl-2 family binding patterns showed that SMS-SAN has a small amount of Bim bound to Bcl-X_L_, whereas NB-1643 has Bim bound exclusively to Bcl-2 [19]. Therefore, testing SMS-SAN with ABT-199 allowed us to evaluate the efficacy of a selective Bcl-2 inhibitor on an NB that may codepend on Bcl-X_L_. NB-1643 and SMS-SAN were very sensitive to single agent ABT-199 with a similar IC_50_ as seen with ABT-737 (IC_50_ 1.5 nM and 5 nM, respectively; Fig. 1a, b). Furthermore, ABT-199 was equipotent as ABT-263 against SMS-SAN, suggesting this NB depends more on Bcl-2. Previous data supports this finding as we have previously shown ABT-737 displaces Bim off of Bcl-2 in SMS-SAN [19]. IMR5, a MYCN amplified HR NB cell line characterized as Mcl-1 dependent [17], was completely resistant to ABT-199 at concentrations as high as 500 nM, similar to that seen with ABT-737 (Fig. 1c; [19]).

**Fig. 2:**
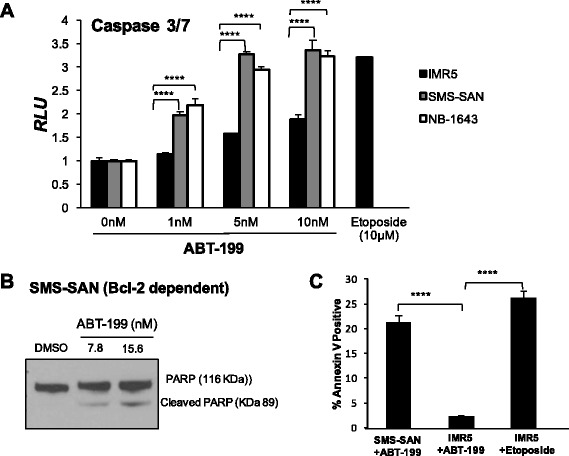
ABT-199 activates the intrinsic mitochondrial apoptosis pathway in Bcl-2 dependent NBs. **a** Following a 24 h exposure to increasing concentrations of ABT-199, both SMS-SAN and NB-1643 (Bcl-2 dependent) show activation of caspases 3 and 7. Mcl-1 dependent cell line, IMR5 does undergo apoptosis in response to the cytotoxic drug, etoposide, but fails to activate caspases 3 and 7 in response to ABT-199. **b** Western blot evidence for downstream PARP cleavage following a 48 h ABT-199 treatment of SMS-SAN. **c** Following 24 h treatment with etoposide or ABT-199, NB cells were analyzed for Annexin V positivity, an indicator of cell membrane externalization of phosphatidylserine and late stage apoptosis

We then evaluated whether cell death induced by ABT-199 proceeds through the intrinsic pathway of apoptosis. Treatment of SMS-SAN with ABT-199 activated both caspases 3 and 7 within 24 h (Fig. 2a). Caspase 3 activation occurs downstream of the mitochondrial outer membrane permeabilization (MOMP), supporting that Bim was released from Bcl-2 to bind to Bax and/or Bak, leading to the release of cytochrome c from the mitochondria and ultimate activation of the effector caspases in SMS-SAN. Apoptosis induction is also supported by the presence of downstream caspase 3 targets such as cleaved PARP in SMS-SAN (Fig. 2b). While caspase 3 and/or 7 increase slightly in IMR5 with ABT-199, this Mcl-1 dependent cell line does not die in response to even higher doses of ABT-199 (Fig. 1c). Caspases can participate in additional cellular processes not associated with apoptotic cell death [20]. Therefore, to validate that caspase 3,7 were activated in SMS-SAN in response to apoptosis induction, we evaluated for late stage apoptosis, phosphatidylserine exposure on the cell surface, by measuring Annexin V by flow cytometry (Fig. 2c). Annexin V did not increase in IMR5 in response to treatment with ABT-199 at the same dose (5 nM) that slightly activated caspases. In contrast, Annexin V did increase in SMS-SAN following ABT-199 exposure (Fig. 2c). We validated that IMR5 can die by apoptosis in response to a cellular stress by showing robust caspase activation as well as Annexin V positivity with a 24 h exposure to etoposide (Fig. 2a, c). These results confirm that Mcl-1 dependent NBs do not die by programmed cell death in response to select Bcl-2 antagonism.

### ABT-199 displaces Bim from Bcl-2 to induce apoptosis in high-risk NB cell lines in vitro

**Fig. 3:**
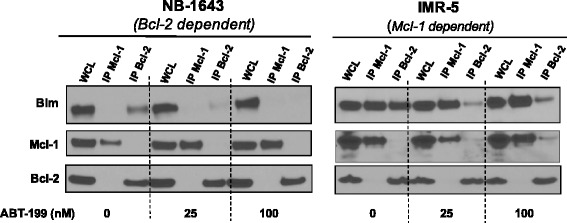
ABT-199 displaces Bim off of Bcl-2, but not Mcl-1 to activate apoptosis. NB cell lines were exposed to ABT-199 for 24 h, followed by co-IP of anti-apoptotic Bcl-2 or Mcl-1 to assess for changes in Bim binding patterns by western blot in response to select Bcl-2 antagonism

The chemical structure of ABT-199 was derived from the backbone of the ABT-263 scaffold and chemically manipulated to selectively bind to the hydrophobic pocket of Bcl-2 [21]. ABT-199 competitively binds to Bcl-2 to either displace or prevent the sequestration of death activator BH3 proteins such as Bim [21]. Given our previous data implicates Bim is the key death effector in HR NB, we evaluated whether ABT-199 induces apoptosis in NB by displacing Bim from Bcl-2. Following treatment of NB cells with ABT-199, anti-apoptotic multidomain proteins, Bcl-2 and Mcl-1, were immunoprecipitated and assessed for Bim binding patterns (Fig. 3). In primary tumors and cell lines, Bim is rarely found bound to Bcl-X_L_ and those that show Bim binding Bcl-xL also have Bim bound to Bcl-2 [19]. In the cell lines tested (NB-1643, IMR5), Bim is not bound to Bcl-X_L_ [19], therefore, we did not immunoprecipitate Bcl-X_L_ in this experiment. In Mcl-1 dependent IMR5, Bim at baseline was bound to both Bcl-2 and Mcl-1. After treatment of IMR5 with ABT-199, Bim moved off of Bcl-2, but remained bound to Mcl-1 given ABT-199 has no affinity for Mcl-1 (Fig. 3). Despite the fact that some Bim is bound to Bcl-2 in IMR5 at baseline, Mcl-1 is the major contributor to apoptosis resistance in IMR5, given it is completely resistant to ABT-199 in in vitro death assays (Fig. 1c). In contrast, Bim is exclusively bound to Bcl-2 in NB-1643 at baseline (Fig. 3) and exposure to ABT-199 leads to displacement of Bim completely off of Bcl-2.

### ABT-199 augments cytotoxic chemotherapy response in NBs in vitro

In tumors such as HR NB, cell stresses like chemotherapy and radiation activate specific BH3-only proteins that then bind to and prime multidomain anti-apoptotic members like Bcl-2. Given the majority of high-risk NB tumors at diagnosis initially respond to cytotoxic chemotherapy then ultimately progress due to acquired chemotherapy resistance, we assessed whether ABT-199 could restore cytotoxic chemotherapy sensitivity in HR NB cell lines***.*** Similar to responses previously seen with ABT-737 and chemotherapy, ABT-199 enhances doxorubicin-induced cell death in Bcl-2 dependent NB cell lines in culture, decreasing the IC_50_ significantly (Table 1). ABT-199 also enhances the cell death effects of the alkylating agent, melphalan, though not to the degree that it does for doxorubicin in SMS-SAN. These chemotherapy augmenting effects by ABT-199 were not seen for the Mcl-1 dependent cell line IMR5, likely due to ABT-199’s selective antagonism for Bcl-2 (Table 1).

**Table 1:** ABT-199 enhances chemotherapy-induced cell death in Bcl-2 dependent NBs. SMS-SAN (Bcl-2 dependent) and IMR5 (Mcl-1 dependent) NB cells were exposed to different combinations of ABT-199 + doxorubicin or ABT-199 + melphalan for 48 h then evaluated by WST-1 for changes in IC_50_ of the combination compared to the cytotoxic chemotherapy given alone. Table numbers represent the average of three biologic experiments

Chemotherapy + ABT-199 in vitro combinations		
	SMS‐SAN (Bcl‐2 Dependent)	IMR-5 Mcl-1 dependent
ABT‐199 dosing (nM)^a^	IC_50_ Doxorubicin (ng/mL)	IC_50_ Doxorubicin (ng/mL)
0	493.6	412.2
1	58.49	401.9
5	15.28	384.9
ABT‐199 dosing (nM)^a^	IC_50_ Melphalan (μM)	IC_50_ Melphalan (μM)
0	6.25	15.29
1	3.3	15.39
5	2.3	14.27

^a^ABT-199 and cytotoxic given concurrently *in vitro* × 48 hours.

### NB xenografts reliant on Bcl-2 for survival have a sustained complete remission to ABT-199 and cyclophosphamide in vivo

**Fig. 4:**
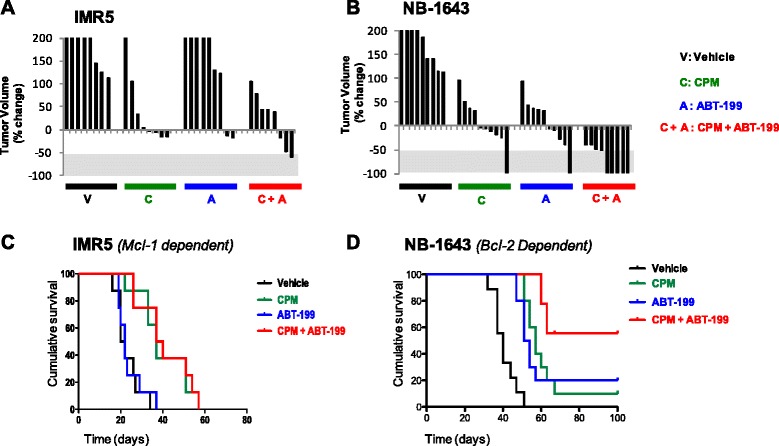
ABT-199 induces complete tumor regression when given in combination with cyclophophosphamide to aggressive murine models of HR NB. NB cell line xenografts representing Bcl-2 dependent (NB-1643) and Mcl-1 dependent (IMR5) subclasses were established in the flank of nu/nu athymic mice. Mice with tumors 150–200 mm^3^ were randomized to receive either intraperitoneal cyclophosphamide (CPM), oral ABT-199 (A), Cyclophosphamide + ABT-199 (CPM + A), or vehicle control (C), as outlined in Methods. Waterfall plot of individual tumor volume as % volume change from tumor volume pre-therapy are depicted for IMR5 (**a**) and NB1643 (**b**) tumors. Negative values define best-response tumor shrinkage with −100 % representing complete regression. Kaplan-Meier curves comparing survival of control (*black*) versus ABT-737 (*blue*), and CPM (*red*) versus CPM + ABT-199 (*green*) in representative xenografts of IMR5 (**c**) and NB1643 (**d**). *p*-values derived using the log-rank test. For NB-1643, *n* = 10 mice per arm; for IMR5, *n* = 9 mice per arm

Most high risk NB patients see two or more cycles of doxorubicin in standard of care upfront therapy and the risk for severe cardiac toxicity increases with cumulative dosing. For this reason, doxorubicin is rarely used for heavily pre-treated patients whose tumors have relapsed. Therefore, while doxorubicin in vitro combination results with ABT-199 were more impressive than melphalan combination results, we chose to test ABT-199 with the alkylator, cyclophosphamide, in vivo given it is the most commonly used cytotoxic for combining and translating novel agents forward into the clinic for HR NB. To adequately compare in vivo responses between previously tested ABT-737 and current ABT-199 treatment of human derived NB cell lines xenografted into mice, we employed the same Mcl-1 dependent (IMR5) and Bcl-2 dependent (NB-1643) cell lines used in prior published assays, using an identical treatment design as previously described ([19] and Methods). Following establishment of a palpable tumor in the flank of athymic nu/nu mice (150–200 mm^3^), the mice (*n* = 8–9/arm for IMR5, 9–10/arm for NB-1643) were segregated to receive vehicle control (normal saline by intraperitoneal injection (IP)), ABT-199 (100 mg/kg oral gavage daily × 14 days), cyclophosphamide (CPM; 75 mg/kg IP twice weekly × 2 weeks), or a combination of ABT-199 and CPM at monotherapy dosing. Results demonstrated that Bcl-2 dependent NB-1643 had an immediate tumor response to both ABT-199 alone and to the combination of ABT-199 and CPM, with measurable tumor regression at the end of treatment (Fig. 4b). IMR5 tumors initially showed some regression to the combination of ABT-199 + CPM as well as to CPM alone (Fig. 4a), suggesting that the CPM and not ABT-199 was the effective component of the combination. While IMR5 xenografts initially responded to CPM, the response was short-lived and all tumors regrew right after completion of therapy, resulting in no statistical survival difference between mice treated with ABT-199 and vehicle, nor the CPM verses CPM + ABT-199 treatment arms (Fig. 4c). In contrast, the Bcl-2 dependent NB-1643 xenografts had significantly prolonged survival after a short monotherapy course of ABT-199 when compared to vehicle control, and underwent sustained complete remissions in 7/9 xenografts treated with the CPM + ABT-199 combination, as far out as 100 days from the brief 14 day treatment. At day 100, the 7 mice remaining were sacrificed given they remained with no detectable tumor. This led to a significant improvement in survival for NB1643 mice treated in the ABT-199 + CPM arm compared to all other arms of the preclinical trial (Fig. 4d). No toxicities were incurred by the mice who all maintained normal weight and behavior in all treatment arms of the experiment. Given the published clinical and preclinical data showing the lack of platelet depression by ABT-199 [13, 14, 21], complete blood counts were not obtained in this trial.

### Bcl-2 dependent NB xenografts that recur following a short course of ABT-199 maintain Bcl-2 dependence

**Fig. 5:**
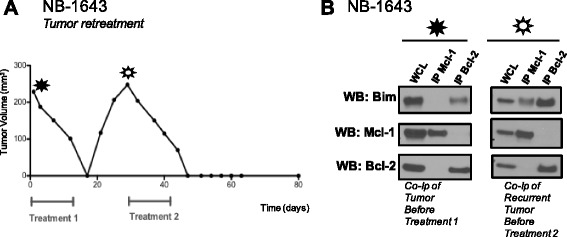
Bcl-2 dependent tumors that recur following ABT-199 therapy maintain Bcl-2 dependence patterns in vivo. 2/9 Bcl-2 dependent tumors treated with ABT-199 + CPM completely regressed but then regrew. **a** One mouse received a second round of ABT-199 + CPM therapy (14 days) that led to a second complete regression of the tumor out to 50 days post second treatment. **b** The other mouse was sacrificed so that the recurrent tumor could be profiled for Bcl-2 dependence patterns by co-IP. Co-IP showed that the xenograft maintained Bim bound to Bcl-2 despite its recurrence. These results suggest that the short duration of treatment led to these two tumor’s growing back

Two out of nine NB-1643 xenografts that initially had complete tumor disappearance in response to ABT-199/CPM combination started to regrow by day 18, four days from completion of therapy. To assess whether the recurrence of tumor was due to an acquired mechanism of Bcl-2 antagonist resistance, such as an adaptation to Mcl-1 dependence, we retreated one mouse and assessed the other tumor for changes in Bim binding by co-IP. The one mouse with recurrent tumor that was subjected to a second cycle of therapy (14 days of the same CPM + ABT-199 regimen once the tumor reached 200 mm^3^ again) completely regressed again with no detectable tumor out as far as day 80 (Fig. 5a). Co-IP of the other recurrent tumor confirmed our in vivo “retreatment” findings that the tumors did not change their Bcl-2 family dependence pattern in response to treatment with ABT-199, as the majority of Bim remained bound to Bcl-2 (Fig. 5b). Noteably, some Bim is now found bound to Mcl-1 in the recurrent tumor (Fig. 5b), alluding to the potential for evolution of Mcl-1 dependent clone in the tumor if ABT-199 treatments are given for a longer duration of time. But given the recurrent tumor still obtained a second complete response to a repeat course of ABT-199/CPM supports that regrowth of the only two tumors that were not cured by this effective combination was likely due to the short duration of treatment and not acquired resistance.

## Discussion

We have previously shown that high-risk NB cell lines derived from human tumors with the poorest prognosis can be functionally dependent on Bcl-2 for survival through Bcl-2’s tonic sequestration of Bim [19]. While the majority of NB cell lines and primary tumors express Bcl-X_L_ protein, Bcl-X_L_ fails to sequester Bim or other activator BH3 proteins to functionally repress programmed cell death. Given the absence of Bcl-X_L_ dependence, we hypothesized that select Bcl-2 antagonism would be equally as potent as ABT-737 in killing NB tumors and eventually result in less hematologic side effects when translated in the clinic. These studies confirmed the potency of select Bcl-2 antagonism in HR NB models.

Given ABT-199 disrupts Bim binding to Bcl-2 at low nanomolar concentrations in vitro, the superiority of the combination regimen over ABT-199 alone in vivo does not indicate that ABT-199 is less effective at inhibiting its target in vivo. Interestingly, the same in vivo experiments we performed with ABT-737 against NB xenografts also showed that ABT-737 was more potent in combination with CPM compared to ABT-737 when given alone. The second remission noted in the recurrent xenograft retreated with ABT-199/CPM suggests that a longer treatment duration (>14 days) with ABT-199 either alone or in combination may reveal the true potency of this drug for the clinic. Further mechanistic studies are warranted to understand the contribution a cytotoxic like cyclophosphamide has in augmenting the impressive tumor regression seen in the *in situ* setting.

We cannot discount the potent effects of cytotoxic chemotherapy in curing the largest portion of patients with HR NB and other solid tumors over the years, frankly at a far higher rate than any targeted therapy has thus far. Thus the results of these combination studies makes an argument that novel pro-apoptotic agents like ABT-199 should not be used as a *substitute* for standard cytotoxics but as *an addition* to chemotherapy, to permit the lowering of cytotoxic dosing to decrease normal cell toxicity while enhancing cytotoxic induced tumor death by reinstating functional apoptotic machinery to the tumor. Given the platelet effects of Navitoclax are not seen in patients clinically treated with ABT-199 supports that this agent has a higher potential to be used in combination with cytotoxic drugs in patients with multiply recurrent NB, who have heavily pretreated marrows with mild thrombocytopenia at baseline.

Like many tumors, Bcl-2 dependence in neuroblastoma cannot be determined by expression-based methods alone but by a functional determination of Bim:anti-apoptotic Bcl-2 protein binding patterns. Functional assays such as mitochondrial BH3 profiling, co-IP and more recently dynamic BH3 profiling can be performed on very small amounts of solid tumor tissue to successfully characterize Bcl-2 dependence patterns leading to apoptosis resistance in chemotherapy refractory solid tumors like NB and others [17, 19, 22]. Further validation of such assays and honing them to be performed in the clinical setting using limited numbers of cells from tumor samples will improve our ability to match patients to the most effective pro-apoptotic therapies like ABT-199 and will further pave the way for using small molecule Bcl-2 antagonists in the clinic for those pediatric NB patients most likely to respond.

## Conclusions

The extreme clinical responses to ABT-199 seen in patients with CLL and AML as well as ongoing clinical trials of ABT-199 in patients with CML sets forth a legitimate role for select Bcl-2 antagonists in the treatment of “liquid” tumors. Solid tumors are more heterogeneous, often having different Bcl-2 anti-apoptotic protein dependence patterns even within the same tumor type. Our work demonstrates on-target potency of ABT-199 at low nanomolar dosing as monotherapy as well as improved cytotoxic efficacy with durable remissions when given in combination with ABT-199 in vivo*.* Thus this preclinical data sets forth a platform to translate ABT-199 and cyclophosphamide combination into a clinical trial, using biologic correlates in the form of a functional assay to determine those HR NB patients most likely to benefit from this novel pro-apoptotic regimen.

## Abbreviations

Bcl-2: B-cell Lymphoma 2
CLL: chronic lymphocytic leukemia
Co-IP: co-imunoprecipitation
CPM: cyclophosphamide
HR NB: high-risk neuroblastoma
Mcl-1: Mantle cell lymphoma 1
